# Molecular Insights Into SARS COV-2 Interaction With Cardiovascular Disease: Role of RAAS and MAPK Signaling

**DOI:** 10.3389/fphar.2020.00836

**Published:** 2020-06-03

**Authors:** Zena Wehbe, Safaa Hammoud, Nadia Soudani, Hassan Zaraket, Ahmed El-Yazbi, Ali H. Eid

**Affiliations:** ^1^Department of Biology, American University of Beirut, Beirut, Lebanon; ^2^Department of Pharmacology and Therapeutics, Beirut Arab University, Beirut, Lebanon; ^3^Department of Experimental Pathology, Immunology and Microbiology, American University of Beirut, Beirut, Lebanon; ^4^Department of Pharmacology and Toxicology, American University of Beirut, Beirut, Lebanon; ^5^Department of Pharmacology and Toxicology, Faculty of Pharmacy, Alexandria University, Alexandria, Egypt; ^6^Department of Biomedical Sciences, College of Health, Qatar University, Doha, Qatar

**Keywords:** severe COVID-19, cardiovascular burden, signaling pathways, RAAS, MAPK signaling

## Abstract

In December 2019, reports of viral pneumonia came out of Wuhan city in Hubei province in China. In early 2020, the causative agent was identified as a novel coronavirus (CoV) sharing some sequence similarity with SARS-CoV that caused the severe acute respiratory syndrome outbreak in 2002. The new virus, named SARS-CoV-2, is highly contagious and spread rapidly across the globe causing a pandemic of what became known as coronavirus infectious disease 2019 (COVID-19). Early observations indicated that cardiovascular disease (CVD) patients are at higher risk of progression to severe respiratory manifestations of COVID-19 including acute respiratory distress syndrome. Moreover, further observations demonstrated that SARS-CoV-2 infection can induce *de novo* cardiac and vascular damage in previously healthy individuals. Here, we offer an overview of the proposed molecular pathways shared by the pathogenesis of CVD and SARS-CoV infections in order to provide a mechanistic framework for the observed interrelation. We examine the crosstalk between the renin-angiotensin-aldosterone system and mitogen activated kinase pathways that potentially links cardiovascular predisposition and/or outcome to SARS-CoV-2 infection. Finally, we summarize the possible effect of currently available drugs with known cardiovascular benefit on these pathways and speculate on their potential utility in mitigating cardiovascular risk and morbidity in COVID-19 patients.

## Introduction

Coronaviruses (CoVs) are single-stranded, positive-sense RNA viruses (26–32 kb) that belong to the *Coronaviridae* family (Su et al., 2016). Common human coronaviruses include HCoV-NL63, -229E, -OC43, and -HKU1 and are usually associated with mild acute respiratory illnesses or “common cold.” In December 2019, clusters of pneumonia cases of unknown etiology were reported in Wuhan City, Hubei Province in China (Huang et al., 2020). Within a few weeks, scientists determined that these mysterious pneumonia cases were caused by a novel coronavirus (CoV) that shares around 79.5% sequence similarity with the SARS-CoV and 96.2% with bat-CoV RaTG13 (Lu et al., 2020; Zhou P. et al., 2020). Therefore, the virus was named SARS-CoV-2 and the disease COVID-19 stands for coronavirus infectious disease 2019. On March 11, 2020, the World Health Organization announced COVID-19 as a pandemic and requested all countries to scale up their emergency response mechanisms (Who, 2020b).

SARS-CoV2 is highly contagious with an average incubation period of 5–6 days (range 1–14) (Who, 2020a). It can be transmitted by droplets generated during coughing or sneezing, close contact, and touching contaminated surfaces (Prevention, 2020). The typical symptoms of COVID-19 are fever, cough, fatigue, and shortness of breath (Who, 2020a). About 15% of SARS-CoV2 positive cases become severe-to-critical with the following complications: pneumonia, acute respiratory distress syndrome, arrhythmia, septic shock, and/or multiple organ dysfunction/failure (Who, 2020a; Zheng et al., 2020).

A relationship between COVID-19 and cardiovascular disease (CVD) is becoming increasingly evident. Indeed, patients with CVD are at a higher risk of developing severe COVID-19 complications, and viral infection might, itself, induce cardiovascular injury. In this review, we provide an overview of the possible pathways that are common to SARS-CoV infections and CVD that might underlie this mutually reinforcing relationship. We also examine the possible modifying effect of some of the available therapies that could confer a protective effect or mitigate the severity of the disease complications.

## Cardiovascular Involvement in COVID-19: A Two-Way Road

### Increased Severity of COVID-19 in Patients With CVD

CVD is a risk factor for the progression of severe disease following lower respiratory tract infection by SARS-CoV or MERS-CoV (Oudit et al., 2009; Alhogbani, 2016). However, many factors complicate the accurate identification of prevalence of CVD in infected patients. Nevertheless, association between existing CVD and increased risk of progression to severe COVID-19 complications was suggested (Liu et al., 2020; Rodriguez-Morales et al., 2020; Zhou F. et al., 2020). The incidence of these comorbidities was higher in patients requiring intensive care admission (ICU) than non-ICU patients. Other studies show that the incidence of CVD in COVID-19 patients is relatively high (Guan et al., 2020; Yang et al., 2020). Moreover, the risk of developing acute respiratory distress or in-hospital death was shown to increase by at least 2-fold for hypertension and over 20-fold for coronary artery disease (Wu et al., 2020; Zhou F. et al., 2020). A report on the incidence of CVDs in the general population in China showed a 23.2% incidence rate for hypertension (Ma et al., 2020). This is close to the rates at which these comorbidities appear among COVID-19 patients. As such, it is likely that CVD patients have an increased severity of COVID-19 complications rather than an increased vulnerability to infection. Indeed, this was shown to be the case in several recent studies (Li et al., 2020; Mehra et al., 2020). Therefore, it is important to identify if and which signalling pathways in CVD may augment SARS-CoV-2 pathogenesis.

### SARS-CoV-2-Induced Myocardial Damage

Accumulating case studies document acute cardiac manifestations in COVID-19 patients, who were previously healthy (Inciardi et al., 2020). Typically, most studies define acute myocardial injury by elevated cardiac troponin I levels (Bansal, 2020; Huang et al., 2020; Zhou F. et al., 2020). High levels of troponin or creatine kinase were noted in a significant fraction of patients diagnosed with COVID-19 (Huang et al., 2020; Wang et al., 2020). Moreover, among patients who were without previous CVD but died following SARS-CoV-2 infection, 11.8% had high levels of troponin indicating myocardial injury and cardiac arrest (Wang et al., 2020). Another study showed that 27.8% of COVID-19 patients had myocardial injury resulting in cardiac dysfunction and arrythmias (Guo et al., 2020). This study highlighted the impact of myocardial injury showing that patients with high troponin levels and no previous history of CVD had a mortality rate of 37.5%, whereas even those with underlying CVD and normal troponin levels demonstrated a better prognosis (13% mortality). Troponin levels were correlated with increases in C-reactive proteins suggesting a tight link between myocardial injury and inflammatory pathogenesis (Guo et al., 2020). Systemic inflammation is intimately related to reduced coronary blood flow and decreased oxygen supply to the heart even in the absence of other risk factors of coronary artery disease (Recio-Mayoral et al., 2009). Yet, myocardial damage induced by direct viral entry through binding to angiotensin converting enzyme-2 (ACE2) on cardiac cells could not be eliminated either (Oudit et al., 2009). Patients who recovered from infections by previous SARS-CoVs reported disruption in blood lipids and blood pressure after 12 years of recovery (Wu et al., 2017). Given the structural similarity between SARS-CoV and SARS-CoV-2, a similar profile could be expected.

### SARS-CoV-2-Induced Endothelial Damage and Intravascular Coagulation

Viral infections are known to activate coagulation cascade, a mechanism thought to be protective as to limit viral spread (Milbrandt et al., 2009; Antoniak and Mackman, 2014). Yet, excessive coagulation could eventually lead to disseminated intravascular coagulation. Contextually, D-dimer and fibrin/fibrinogen degradation products were both higher in COVID-19 patients than controls, with the majority of non-survivals having disseminated intravascular coagulation during their hospital stay (Guan et al., 2020; Han et al., 2020; Tang et al., 2020; Zhou F. et al., 2020). The mechanism behind this activated and accelerated coagulation in COVID-19 patients could be an inflammatory-immunological stimulation likely caused by vascular endothelial damage. Significantly, ACE2 is localized on endothelial cells (Gallagher et al., 2008), and SARS-CoV-2 has recently been shown to cause endothelial cell infections across vascular beds in COVID-19 patients (Varga et al., 2020). On the other hand, endothelial dysfunction and the activation of the clotting cascade are common occurrences in CVD (Lowe and Rumley, 2014; Widmer and Lerman, 2014). As such, a thorough understanding of the signaling pathways common to the pathogenesis of CVD and SARS-CoV-2 infection is necessary to identify crucial sites of crosstalk and direct future investigation of potential therapeutic interventions mitigating both COVID-19 complications in CVD patients as well as short- and long-term viral-induced cardiovascular impairment.

## Molecular Pathways Implicated in the Crosstalk Between CVD and SARS-CoV-2 Pathogenesis

### ACE2, the Renin-Angiotensin-Aldosterone System, and MAPK Pathways

**Figure 1:**
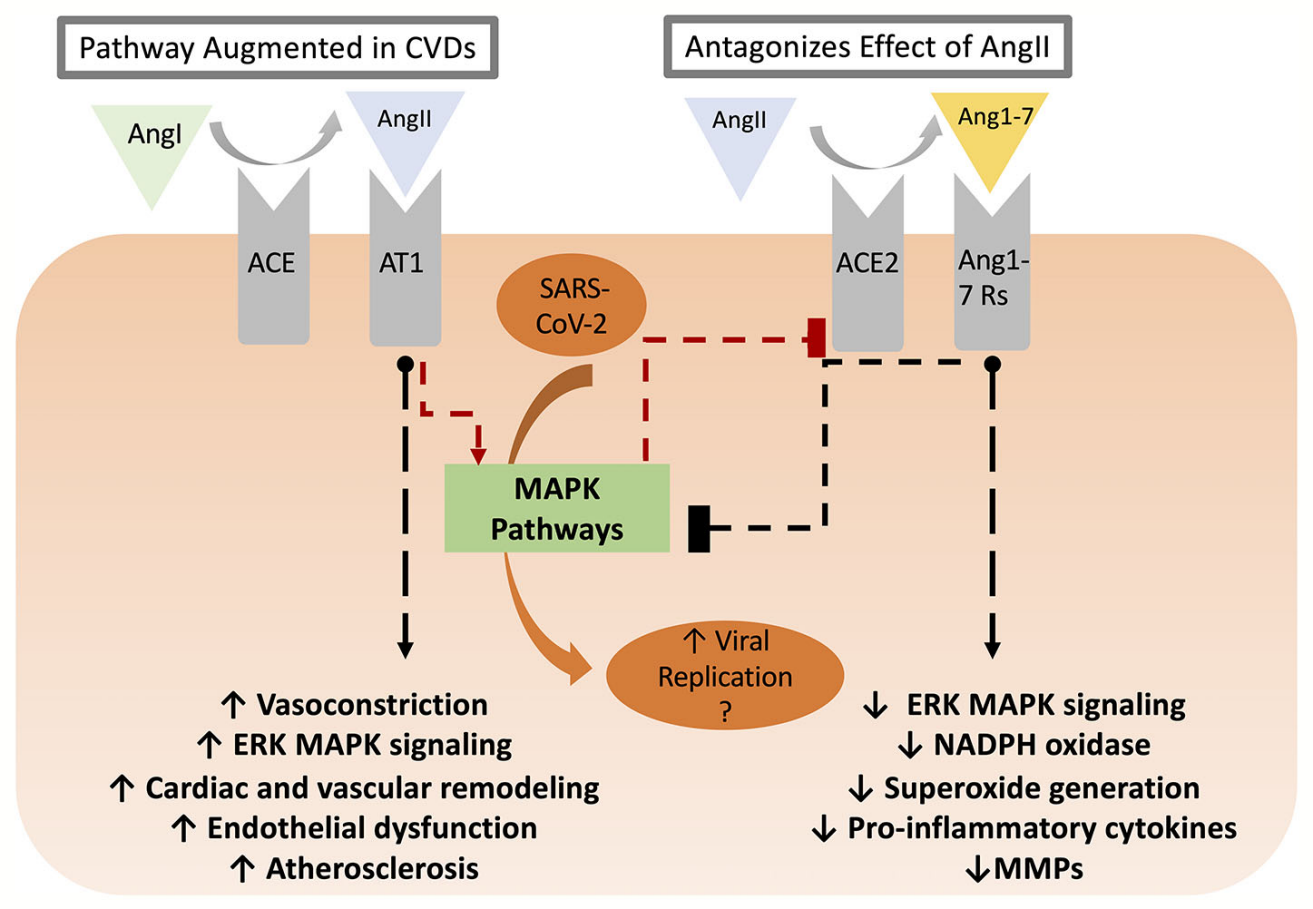
The RAAS balance between AngII/AT_1_-R and ACE2/Ang(1-7)/Ang(1-7)R axes. The former is enhanced in CVD conditions triggering various intracellular pathways *via* MAPK signaling, which ultimately leads to cardiac and vascular remodeling, endothelial dysfunction, and atherosclerosis. ACE2, on the other hand, decreases inflammation by countering the effect of the ACE/AngII/AT_1_-R axis. ACE2/Ang(1-7)/Ang(1-7)R signaling pathway not only ameliorates cellular proliferation, hypertrophy, oxidative stress, and vascular fibrosis but also reduces the activation of the downstream MAPK cascades. AT_1_-R activation triggers p38 MPAK-dependent ACE2 excision and shedding leading to a reduced cell surface expression. As demonstrated in other coronaviruses, SARS-CoV-2 replication might be enhanced as a result of increased MAPK activity downstream of AngII in CVD patients.

**Figure 2:**
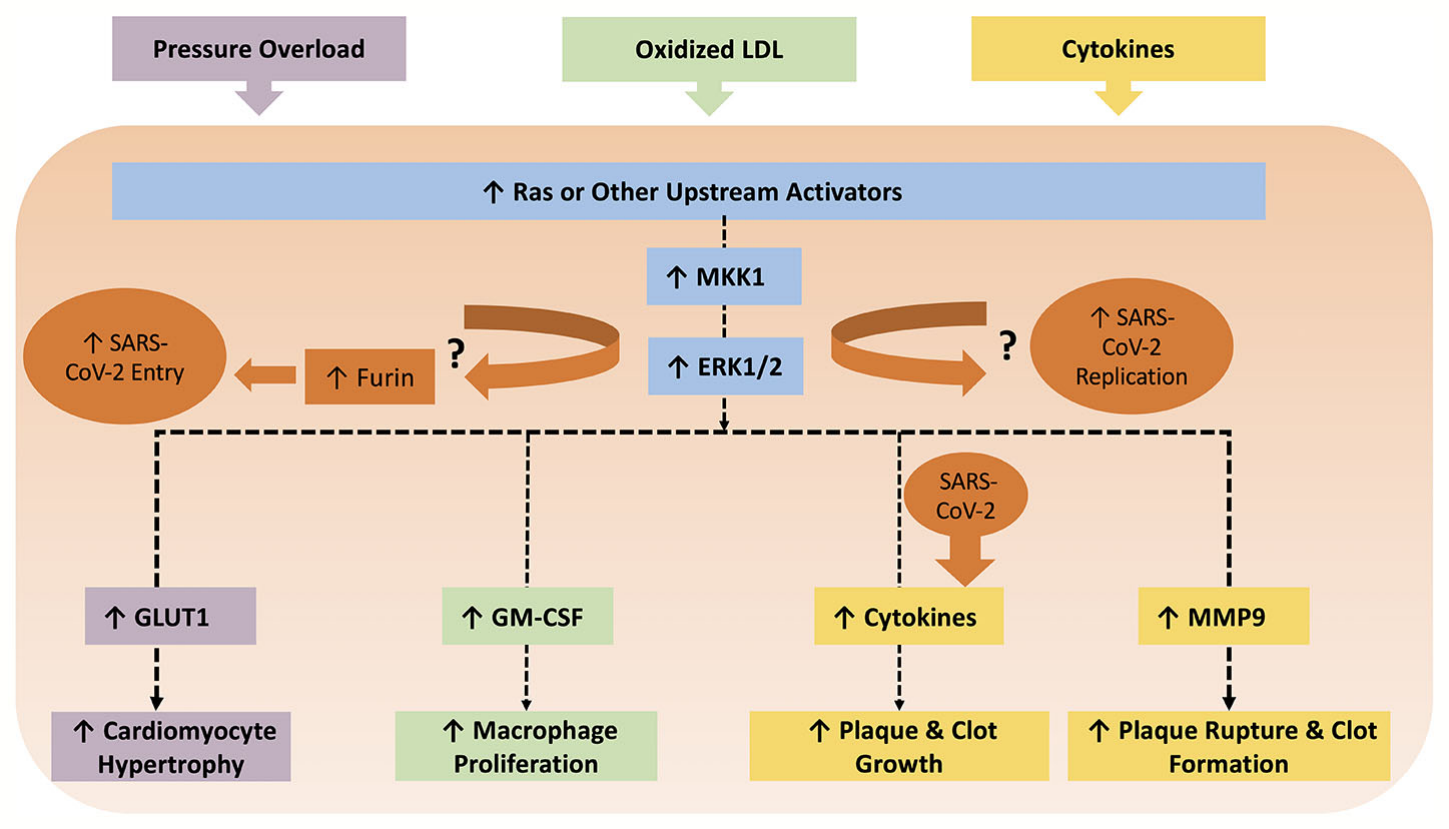
The MAPK pathway can be induced by several triggers in various cells within the context of CVDs. For example, pressure overload can cause hypertrophy in cardiomyocytes *via* the ERK1/2/GLUT pathway. Oxidized LDL triggers macrophage proliferation *via* ERK1/2/GM-CSF, which contributes to the development of atherosclerosis. All cells involved in atherosclerosis can release cytokines in an ERK1/2 dependent manner, which ultimately propel plaque and clot growth. Cytokines can subsequently enhance MMP9 production, which leads to plaque rupture and thrombosis. Meanwhile, coronaviruses have been shown to involve p38 MAPK, JNK, and MKK1/ERK1/2 pathways for viral pathogenesis. MKK1/ERK1/2 pathway also upregulates the protease furin, which is implicated in SARS-CoV-2 entry due to the unique furin-like S1/S2 cleavage site. It is worth examining if inhibition of MKK1/ERK1/2 mitigates production of SARS-CoV-2 viral progeny. On the other hand, SARS-CoV-2 can also amplify production of cytokines, which can worsen existing CVDs.

CVD conditions are typically associated with increased RAAS activity, favoring increased AngII levels (Nehme and Zibara, 2017b). In this regard, AT_1_ receptor activation facilitates various intracellular pathways involved in cardiac and vascular remodeling, endothelial dysfunction, and atherosclerosis (Muslin, 2008; Nehme and Zibara, 2017a). AngII-mediated AT_1_ receptor stimulation activates protein kinases such as mitogen-activated protein kinases (MAPK) (Muslin, 2008; Kawai et al., 2017). On the other hand, Ang(1-7), produced by ACE2-catalyzed cleavage of AngII, is essential in repressing MAPK cascades (Zhang et al., 2014) and decreasing inflammation by keeping a well-balanced RAAS activity, between ACE/AngII pathway and ACE2/Ang(1-7) (Nehme and Zibara, 2017b). While the cardiovascular outcomes of ACE2 activity and Ang(1-7) production have been described in reasonable detail, it remains uncertain as to the nature of the receptor mediating these effects. Early studies reported that mitochondrial assembly 1 receptors (MasR) mediated the AngII/AT_1_R antagonistic effect of Ang(1-7) including PI3k/Akt activation of nitric oxide synthase and NO production, vasodilation, and anti-fibrosis effects (Azushima et al., 2020). Yet, recent studies showed that Ang(1-7)-mediated AT_2_R activation might contribute to the protective effect *via* activation of phosphotyrosine phosphatase and suppression of MAPK activity (Azushima et al., 2020). Moreover, other studies implicated other Mas-related G protein-coupled receptors as mediators of Ang(1-7) effects in the cardiovascular system (Tetzner et al., 2016). Nevertheless, the RAAS and MAPK pathways interaction has many consequences on CVD (summarized in **Table 1** and **Figure 1**) and is implicated in several steps of SARS-CoV-2 pathogenesis. As such, it is important to highlight potential points of hijack by SARS-CoV-2, which are potentially augmented in CVD or could induce *de novo* cardiovascular injury. These points are summarized in **Figure 2**.

**Table 1:** Interactions and examples of cardiovascular consequences of RAAS imbalance and MAPK pathway activation

Contribution of RAAS imbalance to CVD and interaction with MAPK pathways:	-↑AngII→↑mechanisms involving growth factor receptors (e.g., PDGF and TGF-β receptors) →↑ERK activation cardiac and vascular hypertrophy and fibrosis	(Schellings et al., 2006; Ehanire et al., 2015)
	-↑AngII activity in CVD→↓cell surface expression of ACE2 in an AT_1_/p38 MAPK-dependent pathway leading to protein excision and shedding	(Patel et al., 2014; Xu et al., 2017)
↑AngII/AT_1_R pathway→↑MAPK signaling→↑signs of CVD	-ACE2 deletion→↑NADPH oxidase activity, superoxide generation, MAPK signaling and inflammatory cytokine production in the aorta of mice receiving AngII	(Jin et al., 2012)
	-↑ACE2/Ang-(1-7)pathway→↓AngII-induced inflammation in cardiac tissue and in hypothalamic cardiovascular centers, cardiac remodeling, and hypertension	(Grobe et al., 2007; Sriramula et al., 2011)
↑ACE2/Ang(1-7) pathway→↓MAPK signaling→↓ inflammation→amelioration of CVD	-ACE2 overexpression →↓TNF-α, IL-1β, and IL-6 in and ↑anti-inflammatory cytokine IL-10 in autoimmune myocarditis	(Sukumaran et al., 2011)
Contribution of MAPK signaling to CVD	-Erk1/2. JNK, and p38 MAPK activation →↑cardiomyopathic remodeling	(Wang, 2007)
	-Pressure overload→↑Ras/c-RAF/MKK1/ERK1/2 pathway→cardiac hypertrophy, ↑cardiomyocyte size, diastolic dysfunction, and myofibril disarray	(Hunter et al., 1995)
	-ERK1/2 →↑ c-Fos →↑ GLUT1 transporter expression in hypertrophic and ischemic heart	(Babu et al., 2000) (Santalucia et al., 2003) (Shao and Tian, 2015)
	-MAPK pathway activation→progressive endothelial dysfunction	(Huang et al., 2012)
	-Oxidized LDL→↑ MAPK pathway activation→↑GM-CSF→↑macrophage infiltration of atherosclerotic plaques	(Proctor et al., 2007; Muslin, 2008)
	-↑MAPK pathway activation→↑interleukins and TNFα→↑atherosclerotic lesion progression	(Bachstetter and Van Eldik, 2010; De Souza et al., 2014)
	-TNFα→↑ERK1/2 activity→↑MMP9 →↑fibrotic lesion formation	(Cheng et al., 2012; Zhang et al., 2017)
	-Viral infection→↑IL-1, TNFα, IL-6→↑p38 MAPK and JNK →↑viral cardiomyopathy	(Monsuez et al., 2007; Wang et al., 2014; Yue-Chun et al., 2016; Niu et al., 2017)

PDGF, platelet-derived growth factor; TGF, transforming growth factor; TNF, tumor necrosis factor; MKK, MAPK kinase; GLUT, glucose transporter; LDL, low-density lipoprotein; MMP, matrix metalloproteinase.

### ACE2 and Viral Binding to Host Cells: Impact on CVD and Myocardial Injury

Current knowledge suggests that the membrane bound spikes of coronaviruses, known as S glycoproteins, are solely responsible for enabling host cell attachment and entry (Fehr and Perlman, 2015). SARS-CoV-2 gains host cell entry *via* ACE2 expressed on pulmonary alveolar epithelial cells (Letko et al., 2020). Recently, it has been suggested that two S glycoproteins can bind to a single ACE2 homodimer (Yan et al., 2020). Binding occurs *via* direct interaction of the ACE2 peptidase domain and the ACE2-binding domain on the S1 subunit of the S protein. ACE2 is also expressed on the surface of epithelial cells in lungs, heart, kidneys, and intestine, facilitating viral entry to these tissues and hence might underlie the multi-organ dysfunction seen with COVID-19 (Zhang et al., 2020). However, for proper entry into the cell, the S protein must be primed by host cell proteases at a second cleavage site on the membrane embedded S2 subunit (Bosch et al., 2003; Lu et al., 2015; Coutard et al., 2020). Specifically, cleavage at the S2' cleavage site exposes S2 domains, like the fusion peptide and internal fusion peptide, and enables them to partition into the membrane and facilitate entry (Lu et al., 2015). A recent study showed that compared to the proteases required for viral entry, ACE2 has a lower expression level in different epithelial cell types indicating that it is likely to be the rate limiting step in viral entry (Sungnak et al., 2020).

An early study on human ventricular tissue from heart failure patients showed an increased expression and activity of ACE2 (Zisman et al., 2003). Correspondingly, a recent study showed that heart failure patients with COVID-19 had increased levels of ACE2 placing them at higher risk of severity of symptoms (Chen et al., 2020). This might result from increased viral entry due to a higher ACE2 expression level. However, it may also be due to pathways triggered by the virus that can exacerbate CVD symptoms. Interestingly, the same study indicated that cardiac pericytes demonstrated a much higher level of ACE2 expression than cardiac myocytes raising the possibility that the initial cardiac injury might be microvascular. Previously, it was demonstrated that the endothelial cell expression of ACE2 provides a possible route of entry to SARS-CoV once it enters the blood (Ding et al., 2003). The same may be expected of SARS-CoV-2. Viral entry and inflammation could upregulate tissue factor expression on the cell surface, promoting the blood coagulation cascade (Witkowski et al., 2016). Fibrinoid necrosis and thrombotic events will therefore follow in microvessels leading to disseminated intravascular coagulation. Thus, endothelial injury exacerbates the immuno-inflammatory condition by inducing coagulation abnormalities (Vallet and Wiel, 2001). As such, supply of soluble ACE units could, in principle, reduce the infectious burden and rescue the tissues/organs from the detrimental outcome of SARS-CoV-2 infection. Indeed, a recent study showed that treatment with clinical grade human recombinant ACE2 reduced viral recovery from infected cell culture systems (Monteil et al., 2020). The same study also showed that soluble ACE2 treatment inhibited SARS-CoV-2 infection in blood vessel and kidney organoids.

On the other hand, binding of SARS-Cov-2 to ACE2 could eventually deplete ACE2 levels. Indeed, prior studies on the earlier SARS-CoV strain showed that pulmonary and myocardial infections were associated with marked reduction of ACE2 expression (Kuba et al., 2005; Oudit et al., 2009). This would inhibit the protective effects of the ACE2/Ang(1-7) pathway, offsetting the RAAS balance and causing potential exacerbation of respiratory symptoms and cardiovascular complications (Sun et al., 2020). In fact, ACE2 downregulation induced by viral spike glycoprotein injection led to increased pulmonary failure *in vivo* (Kuba et al., 2005). Therefore, ACE2 plays two contradictory roles in patients with cardiovascular/cardiometabolic diseases inflicted with COVID-19. On one hand, ACE2 serves as a gate for SARS-COV-2 infection and on another it protects against CVD as well as the increased production of inflammatory cytokines.

### MAPK, Viral Entry, and the Hijacked Proteases

Non-endosomal SARS-CoV-2 uptake is mostly associated with the transmembrane serine protease TMPRSS2, in contrast to the less frequently used endosomal cysteine proteases cathepsin B and L (CatB/L) (Hoffmann et al., 2020). Interestingly, a unique furin-like protease recognition sequence at the S1/S2 cleavage site in SARS-CoV-2 is absent from the other members of the coronavirus family (Coutard et al., 2020). Furin is a serine protease and considered a proprotein convertase, which transforms protein precursors into active forms upon cleavage. Its function extends to a variety of proteins including growth hormones, cytokines, and surface glycoproteins of viruses (Coutard et al., 2020). It was previously established in another class of viruses that the presence of furin cleavage sites is associated with more pathogenicity as compared to cleavage by trypsin proteases (Kido et al., 2012). Incidentally, atherosclerotic patients demonstrate elevated levels of furin, particularly in foam cells of plaques (Zhao et al., 2018). Furthermore, this protease is regulated by ERK pathways under inflammatory conditions (Ventura et al., 2017). In fact, ERK1 is essential for maintaining activity of furin and has been implicated as a potential therapeutic target to combat the pro-inflammatory effects of TGF-β. Because ERK1 activity is heightened in CVD patients, it is likely that furin activity is also increased. Therefore, it is important to consider the role of elevated furin expression/function in the virulence of SARS-CoV-2.

### MAPK and Viral Pathogenesis

Not only is the Raf/MEK1/2/ERK1/2 pathway heavily implicated in CVDs (**Table 1**), but it is also indispensable for effective viral replication (Li et al., 2019). Specifically, SARS-CoV spike and nucleocapsid proteins were reported to trigger ERK1/2 phosphorylation with subsequent induction of pro-inflammatory pathways including increased cyclooxygenase-2 expression and IL-8 release (Chang et al., 2004; Mizutani et al., 2004a; Yan et al., 2006; Liu et al., 2007). Significantly, while some report no change in SARS-CoV infected cell death after ERK1/2 inhibition (Mizutani et al., 2004a), inhibition of the MEK1/2/ERK1/2 pathway has been shown to significantly impair coronavirus replication in mice (Cai et al., 2007). Similarly, JNK activity increased in cells exposed to SARS-CoV spike and nucleocapsid proteins (Mizutani et al., 2004b; Surjit et al., 2004; Liu et al., 2007). Interestingly, JNK inhibition precluded the development of persistent SARS-CoV infections (Mizutani et al., 2005). Furthermore, SARS-CoV infection was shown to activate p38 MAPK and the downstream signaling possibly leading to cell death (Mizutani et al., 2004b; Surjit et al., 2004; Kopecky-Bromberg et al., 2006). Contextually, p38 MAPK inhibition reduced human coronavirus HCoV-229E viral replication in human lung epithelial cells (Kono et al., 2008). Moreover, regardless of the cell type or strain of the virus, MEK1/2 inhibition diminishes the production of viral progeny (Cai et al., 2007). As previously discussed, many MAPK pathways are already significantly upregulated in CVD possibly downstream of the AngII/AT_1_ pathway activation. The chronic nature and gradual development timeframe of these diseases might argue that the crosstalk among increased MAPK signaling cascades and SARS-CoV-2 pathogenesis leads to increased infection severity and complications in patients with established CVD rather than being involved in viral-triggered cardiovascular involvement. Nevertheless, viral-induced myocardial injury and endothelial involvement are thought to include a strong inflammatory component as well as an offset of the RAAS balance away from ACE2/Ang(1-7) arm, both of which interacts closely with MAPK pathways (**Table 1**).

## Pharmacological Interventions Modifying Pathways Relevant to Viral Infection

Accumulating evidence shows that cardiovascular risk reduction, especially on the long term, involves modification of RAAS and MAPK signalling pathways, regardless of the particular intervention applied. For instance, exercise training reduces p38 MAPK and ERK activities decreasing the incidence of heart failure (Nagata and Hattori, 2011) and ameliorates the AngII/Ang(1-7) imbalance in hypertensive rats (Ren et al., 2016). Similarly, dietary restriction reduced ERK activation (Xie et al., 2007; Castello et al., 2011) and decreased serum ACE activity (Harp et al., 2002). In this section, we provide an overview of the activity of some of the available therapeutic agents or classes with cardiovascular benefit. We focus on drug classes that can potentially modulate ACE2 and/or MAPK signalling pathways, hence decreasing cardiovascular risk on long-term use. We will then speculate on the possible impact of these agents to reduce risk of progression to severe COVID-19 in CVD patients. As will be seen with some of these agents, clinical and research interest in their use for the mitigation of acute complications of SARS-CoV-2 infections has also emerged.

### ACEIs/ARBs

Antihypertensive drugs such as angiotensin converting enzyme inhibitors (ACEI) and AngII receptor blockers (ARBs) gained interest in the context of treating patients with COVID-19. Several studies reported conflicting findings regarding the effects of ACEI/ARBs on ACE2 levels. Some of which reported an increase in ACE2 levels that could be explained based on inhibition of AT_1_ receptor-mediated ACE2 shedding (Patel et al., 2014; Xu et al., 2017), whereas others argued for a role for higher levels of AngII detected after treatment with ARBs, a case of higher substrate availability leading to an increase in expression of the linked enzyme (Dickstein et al., 1994; Gavras and Gavras, 1999; Ferrario et al., 2005; Soler et al., 2009; Sukumaran et al., 2011; Furuhashi et al., 2015; Esler and Esler, 2020). Yet, other studies have documented no effect of ARBs in this regard (Campbell et al., 2004; Burchill et al., 2012). While it could be postulated that treatment with ACEI/ARBs increasing ACE2 levels might increase risk of SARS-COV-2 infection severity, recent studies showed that continuation of these drugs in COVID-19 patients was not associated with such risk (Mancia et al., 2020; Mehra et al., 2020; Reynolds et al., 2020). On the other hand, ACE2 internalization was shown to be dependent on heterodimerization with AT_1_-R and subsequent activation of AT_1_-R with AngII (Deshotels et al., 2014). Hence, even if these drugs increase ACE2 expression, they could confer a protective effect *via* suppression of ACE2-mediated viral internalization (Deshotels et al., 2014) by blocking AT_1_-R in case of ARBs or reducing the availability of AngII in case of ACEIs. Moreover, reduced induction of MAPK pathways triggered downstream of AT_1_-R might contribute to a less severe viral pathogenesis. Along these lines, a study showed that ACE2 knockout exacerbated severe acute respiratory failure following acid aspiration in mice that was rescued by ARB or recombinant ACE2 treatment (Imai et al., 2005). In this context, elevated systemic AngII in CVD patients could underlie higher risk of infection and further complications as discussed previously. Nevertheless, the studies that reported lack of increased risk with ACEI/ARB use were not designed to detect potential benefit; thus, more focused prospective studies are required to answer this question. On this basis, losartan, an ARB, is now being incorporated in two clinical studies investigating possible reduction of lung injury in patients with COVID-19 (clinicaltrials.gov/ct2/show/NCT04311177, clinicaltrials.gov/ct2/show/NCT04312009).

### Statins

Statins are HMG-CoA reductase inhibitors used for treatment of CVD associated with dyslipidemia. Aside from their hypocholesterolemic effect, statins have a proven anti-inflammatory effect. They have been proposed as an adjunctive therapy in influenza virus infection (Arabi et al., 2020). Simvastatin improved survival in patients with hyper-inflammatory acute respiratory distress syndrome (Calfee et al., 2018). Specifically, atorvastatin attenuated NF-κB signaling within 24 h of MERS-CoV infection (Yuan, 2015). Similarly, atorvastatin showed an anti-inflammatory effect that was dose- and time-dependent effectively attenuating NF-kB ultimately leading to decreased synthesis of inflammatory cytokines and chemokines (Chansrichavala et al., 2009). Significantly, statins, *via* activating Akt signaling, enhance endothelial junction integrity and reduce plasma leakage and acute lung injury (Fedson, 2016). Moreover, rosuvastatin reduced AngII production, AT1 receptor expression, and Erk activation and upregulated ACE2 in rats following vascular injury (Li et al., 2013). Taken together, these observations suggest that CVD patients undergoing statin treatment might benefit from a mitigated basal and SARS-CoV-2-induced inflammatory reaction and hence a reduced risk of progression to severe COVID-19.

### SGLT-2 Inhibitors

Sodium-glucose cotransporter type 2 (SGLT-2) inhibitors have been approved to reduce major cardiovascular events in patients with diabetes though direct mechanisms not necessarily related to their hypoglycemic effects (Kaplan et al., 2018; Scheen, 2018). SGLT-2 inhibitors were found to induce a modest increase in plasma levels of aldosterone and AngII, but still within the low range typical in diabetes, as well as an increase in ACE2, ACE, and angiotensinogen (Kawanami et al., 2017). When administered with ACEI or ARBs, the effect of SGLT-2 inhibitors will shunt the RAAS activation towards ACE2 (Kawanami et al., 2017), thus significantly increasing the beneficial effects of Ag(1–7) leading a vasodilatory, anti-inflammatory, and antioxidative effect in the heart and kidneys. More research is needed to evaluate the possible beneficial use of SGLT-2 inhibitors with ARBs or ACEIs on the cardiovascular and metabolic events in COVID-19.

### Aldosterone Receptor Antagonists

Eplerenone, an aldosterone receptor antagonist, is recommended for the treatment of hypertension, left ventricular hypertrophy, and congestive heart failure (Jewell et al., 2006). Patients treated with eplerenone had lower morbidity and mortality due to CVD, a protective effect thought to be independent on blood pressure. Aldosterone induces cell growth and differentiation through activation of Erk and downstream signaling including NF-κB, suggesting a protective effect of eplerenone *via* inhibition of Erk (Kobayashi et al., 2006). In addition, aldosterone receptor blockade improves cardiac function and remodeling *via* increasing eNOS expression and Akt-mediated phosphorylation (Kobayashi et al., 2005). This raises the possibility for a beneficial effect of eplerenone or other aldosterone receptor antagonists in reducing risk of severe COVID-19 infection in CVD patients or to mitigate the cardiovascular burden of SARS-CoV-2 infection.

### Tocilizumab

Interest in the use of tocilizumab, a monoclonal antibody against IL-6, in COVID-19 patients has emerged especially in those at risk of exaggerated inflammatory response (Luo et al., 2020). In the latter study, about two-thirds of the patients with elevated serum IL-6 levels had co-morbid CVD. Treatment with tocilizumab, originally approved for treatment of rheumatoid arthritis, demonstrated several cardiovascular effects. Tocilizumab led to an increased total cholesterol, low density lipoprotein, and high-density lipoprotein levels increasing the risk of dyslipidemia (Singh et al., 2010). Yet other studies reported a strong reduction in the incidence of CVD with tocilizumab treatment despite the change in lipid profile (Rao et al., 2015). It could be argued that high IL-6 levels induce cardiac fibrosis and increase myocardial infarction and CVDs, a pathway linked to aldosterone activity (Chou et al., 2018), thus justifying a positive cardiovascular impact of tocilizumab treatment. As such, more studies are needed to clarify the possible modulatory effect exerted by IL-6 antagonism in COVID-19 patients, and its impact on the intersecting pathways leading to respiratory and cardiovascular complications.

## Conclusions and Future Directions

Whereas knowledge of the exact mechanisms underlying the pathogenesis and the sequelae of SARS-CoV-2 infection is still emerging, the available evidence could be perceived to form a framework implicating RAAS and MAPK pathway imbalances in the observed association between COVID-19 and cardiovascular dysfunction. On the one hand, predominance of the AngII/AT_1_ receptor arm of RAAS as well as increased MAPK activity in CVD could enhance viral pathogenesis and thus predispose the patients for increased COVID-19 severity. While on the other hand, viral activity including cell entry, viral replication, and induction of inflammatory response might also trigger the same pathway imbalances inducing or exacerbating cardiovascular damage. Despite the presumed efficacy of some of the available therapeutic tools in interrupting this vicious cycle, significant investigation is needed to direct evidence-based clinical use. Moreover, a large window of opportunity exists for the identification and design of selective tools to interfere with these pathways in COVID-19 patients with CVD risk.

## Author Contributions

AE-Y and AE developed the idea and the review framework. ZW, SH, HZ, and NS wrote the first draft of the manuscript. All authors contributed to corrections and adjustment of subsequent iterations of the manuscript. All authors approve and agree with the content.

## Funding

This paper was supported by an AUM-FM MPP grant number 320148 to AE-Y and grant number 320133 to AE.

## Conflict of Interest

The authors declare that the research was conducted in the absence of any commercial or financial relationships that could be construed as a potential conflict of interest.
